# Integrated analysis using ToppMiR uncovers altered miRNA– mRNA regulatory networks in pediatric hepatocellular carcinoma—A pilot study

**DOI:** 10.1002/cnr2.1685

**Published:** 2022-07-20

**Authors:** Senyo S. Whyte, Rebekah Karns, Kyung‐Won Min, Jung‐Hyun Cho, Sanghoon Lee, Charissa Lake, Alexander Bondoc, Je‐Hyun Yoon, Soona Shin

**Affiliations:** ^1^ Division of Pediatric General and Thoracic Surgery Cincinnati Children's Hospital Medical Center Cincinnati Ohio USA; ^2^ Division of Gastroenterology, Hepatology & Nutrition Cincinnati Children's Hospital Medical Center Cincinnati Ohio USA; ^3^ Department of Biology Gangneung‐Wonju National University Gangneung Republic of Korea; ^4^ Department of Biochemistry and Molecular Biology Medical University of South Carolina Charleston South Carolina USA; ^5^ Department of Surgery University of Cincinnati College of Medicine Cincinnati Ohio USA

**Keywords:** miRNA, pediatric hepatocellular carcinoma, RNA sequencing, ToppMiR

## Abstract

**Background:**

Pediatric hepatocellular carcinoma (HCC) is a group of liver cancers whose mechanisms behind their pathogenesis and progression are poorly understood.

**Aim:**

We aimed to identify alterations in the expression of miRNAs and their putative target mRNAs in not only tumor tissues of patients with pediatric HCC but also in corresponding non‐tumorous background livers by using liver tissues without underlying liver disease as a control.

**Methods and results:**

We performed a small‐scale miRNA and mRNA profiling of pediatric HCC (consisting of fibrolamellar carcinoma [FLC] and non‐FLC HCC) and paired liver tissues to identify miRNAs whose expression levels differed significantly from control livers without underlying liver disease. ToppMiR was used to prioritize both miRNAs and their putative target mRNAs in a gene‐annotation network, and the mRNA profile was used to refine the prioritization. Our analysis generated prioritized lists of miRNAs and mRNAs from the following three sets of analyses: (a) pediatric HCC versus control; (b) FLC versus control; and (c) corresponding non‐tumorous background liver tissues from the same patients with pediatric HCC versus control. No liver disease liver tissues were used as the control group for all analyses. Many miRNAs whose expressions were deregulated in pediatric HCC were consistent with their roles in adult HCC and/or other non‐hepatic cancers. Our gene ontology analysis of target mRNAs revealed enrichment of biological processes related to the sustenance and propagation of cancer and significant downregulation of metabolic processes.

**Conclusion:**

Our pilot study indicates that alterations in miRNA–mRNA networks were detected in not only tumor tissues but also corresponding non‐tumorous liver tissues from patients with pediatric HCC, suggesting multi‐faceted roles of miRNAs in disease progression. Our results may lead to novel hypotheses for future large‐scale studies.

## 1 INTRODUCTION

Hepatocellular carcinoma (HCC) is rare in children, yet the second most common pediatric primary liver cancer with an annual incidence of 0.8 cases per million children and 1.5 cases per million adolescents in the United States.^1^ Despite its dismal prognosis, research of pediatric HCC has been hampered due to not only its rarity but also diverseness in respect to etiology, histology, and clinical characteristics.^1^, ^2^ In contrast to adult HCC mostly accompanied by cirrhosis, up to 50% of pediatric HCC cases are sporadic without a history of liver disease and can arise from a non‐cirrhotic liver.^3^ Fibrolamellar carcinoma (FLC) is a sporadic subtype of HCC diagnosed in adolescent and young adults harboring the DNAJB1::PRKACA fusion kinase for most cases.^4^ In addition, various types of genetic, infectious, vascular, metabolic, and cholestatic conditions are risk factors for non‐FLC pediatric HCC with underlying disease.^3^

The mechanisms behind pediatric HCC are poorly understood, and while the miRNome of adult HCC is relatively well‐characterized,^5^ scant data exist on the miRNA profiles in pediatric HCC. The current study aimed to perform integrated analysis of miRNA and mRNA profiles in pediatric HCC and grouped these tissues into three pairs—tumor tissues (which include both FLC and non‐FLC pediatric HCC), FLC, and corresponding non‐tumorous background liver tissues. To identify molecular alterations affecting background livers in addition to tumors, we focused on determining differentially expressed miRNAs in these tissues as compared to liver tissues from subjects with no liver disease. Since target prediction tools solely based on the complementarity of miRNAs and mRNAs do not incorporate the potential functional impacts of the miRNA–mRNA interactions, we then used ToppMiR, a web‐based analytical workbench, to generate a ranked list of miRNAs with the most functionally relevant potential interactions with mRNA transcripts.^6^

Our analysis revealed alterations of numerous miRNA–mRNA axes involved in several biological processes in both pediatric HCC as well as corresponding non‐tumorous background livers, highlighting the potential roles of miRNAs in multiple steps of pediatric HCC development.

## 2 METHODS

### 2.1 Clinical samples

Tumors from patients with HCC, corresponding non‐tumorous background liver tissues, and livers from subjects without liver disease as diagnosed *by the institutional pathologists* were analyzed. Surgical specimens from the Cincinnati Children's Hospital Medical Center (CCHMC) and archived specimens from the Discover Together Biobank were included in the current study and informed consent forms were obtained according to the Institutional Review Board‐approved protocol. The study conforms to the recognized standards (US Federal Policy for the Protection of Human Subjects; Declaration of Helsinki). Clinical characteristics of the samples are described in Table 1. Fourteen samples were initially used for generating mRNA and miRNA expression profiles; technical outliers identified through principal component analysis (PCA) were excluded. The list of samples that were analyzed after excluding outliers is provided in Table S1.

**TABLE 1:** Clinical characteristics

Tumors and paired non‐tumorous livers						
Tumor	ID		Liver disease	Chemo	Age (years, mean ± SD)	Gender (M, F)
	Tumor	Liver				
Non‐FLC	C2‐T	C2‐BG	‐	Post	10.3 ± 5.5	(1, 2)
	C10‐T	C10‐BG	Alagille syndrome	Pre		
	C14‐T	C14‐BG	Abernethy syndrome	Pre		
FLC	C1‐T	C1‐BG	‐	Post	16.3 ± 1.2	(0, 3)
	C5‐T	not available	‐	Pre		
	C13‐T	not available	‐	Pre		

Control livers without liver disease			
ID	Source	Age (years, mean ± SD)	Gender (M, F)
N1	Donor liver	16.3 ± 5.9	(1, 3)
N2	Autopsy		
N3	Autopsy		
N4	Autopsy		

*Note*: Technical outliers identified through principal component analysis (PCA) were excluded and the list of samples included in the analysis is provided in Table S1.

Abbreviation: FLC, fibrolamellar carcinoma.

### 2.2 RNA sequencing

For gene expression profiling, total RNA extracted from frozen tissues was processed using Illumina TruSeq Stranded Total RNA with Ribo‐Zero Library Prep per manufacturer's standard protocol and sequenced using Illumina NovaSeq6000 S (150 bp paired‐end). HISAT2 was used for mapping trimmed reads to HG19.^7^ StringTie was used for the assembly of transcripts.^8^ For miRNA sequencing, small RNAs purified from 2 μg of total RNA using a 15% denaturing polyacrylamide gel were barcoded using adapters, and ligated small RNAs were purified, reverse transcribed and amplified by polymerase chain reaction using the following primers: forward, AATGATACGGCGACCACCGACAGGTTCAGAGTTCTACAGTCCGA; reverse, CAAGCAGAAGACGGCATACGA. cDNA libraries were sequenced using Illumina HiSeq 2000. Cutadapt was used to trim adapters.^9^ Trimmed reads were mapped on miRNA precursors using SHiMPS aligner/miRBase^10^, ^11^ and the reads associated with mature miRNAs were counted.

### 2.3 Data analysis

Data were log2‐transformed and baselined to the median of all samples. All detected transcripts and miRNAs were tested for differential expression using a moderated *t* test (significance cutoff false discovery rate [FDR] <0.05). Hierarchical clustering and PCA analyses were performed for the identification of sample cluster patterns and transcript/miRNA clusters. ToppMiR was used to identify functionally significant miRNAs and their corresponding target mRNAs as described previously.^6^ Briefly, differentially expressed miRNAs were ranked based on their connectivity to putative target mRNAs, which integrates not only the target predictions but also the relative importance of these targets in the context of gene‐annotation networks. ToppMiR uses seven different sources of miRNA–mRNA predictions: PicTar,^12^, ^13^, ^14^, ^15^ mirSVR,^16^, ^17^ TargetScan,^18^, ^19^, ^20^, ^21^ MSigDB,^22^, ^23^ PITA,^24^ and experimentally validated miRecords^25^ and miRTarbase.^26^ Differentially expressed mRNA transcripts whose level changed inversely with miRNAs were used as putative target sets to define the miRNA–mRNA interactions and facilitate prioritization. While some of the prioritized miRNAs were not the dominant form according to the miRBase database,^11^ we included all top‐ranked miRNAs in the subsequent ontology analysis. Ontological analysis of confirmed target mRNAs with differential regulation was performed using ToppGene, ToppCluster, and Cytoscape.^27^, ^28^, ^29^

## 3 RESULTS

### 3.1 Samples used for RNA sequencing

Fourteen total samples consisting of six tumors from pediatric patients with HCC (henceforth referred to as “Tumor,” which includes both FLC and non‐FLC tumor samples), four paired non‐tumorous liver tissues (=“BG”), and four control liver tissues from patients without liver disease (=“Control”) were initially subjected to both mRNA and miRNA sequencing (Table 1). After quality assessment of miRNA profiles, selected samples were subjected to subsequent analysis (Table S1). Differentially expressed miRNAs were analyzed for all the possible comparisons. The following three sets of comparisons identified differentially expressed miRNAs between groups with statistically significant changes: “Tumor versus Control,” “FLC versus Control,” and “BG versus Control” (Tables S2–S4). Other comparisons of miRNAs such as “Tumor versus BG” or “FLC versus non‐FLC” did not achieve significance. Hierarchical clustering and PCA indicated a clear separation of Control from other groups (Figure S1).

### 3.2 miRNA–mRNA profiling of tumor tissues revealed several deregulated biological processes in pediatric HCC

Our analysis identified 27 upregulated miRNAs in Tumor versus Control, and 30 miRNAs were downregulated (Fold Change>2, FDR <0.05) (Table S2). To understand the role of differentially regulated miRNAs in pediatric HCC, we performed ontology analysis. ToppMiR was used to rank the potential target mRNAs found in publicly available RNA‐seq data based on their nominal *p* values and to prioritize them according to gene annotations that indicate their potential to impact a given biological system.^6^ The list of differentially expressed mRNAs in Tumor versus Control (Table S5) was integrated into the pipeline to further refine the analysis. Differentially expressed miRNAs were then ranked based on their connectivity to mRNA transcripts, with the most potentially impactful transcript/miRNA ranked 1. miRNAs with significant connectivity to the mRNA test set were then used to rank mRNAs based on the number and specificity of miRNA targeting. The use of ToppMiR to analyze RNA‐seq data thus generated two separate rankings for both miRNAs (Table 2) and target mRNAs, which can then be used to draw inferences about the most biologically impactful consequences of these molecules. Table S6 describes the lists of downregulated target mRNAs of upregulated miRNAs and upregulated target mRNAs of downregulated miRNAs. While some of the prioritized miRNAs were not the dominant strand according to the miRbase database,^11^ we included all forms in the subsequent ontology analysis, since recent evidence suggests that both strands can be functional.^30^

**TABLE 2:** Top‐ranked miRNAs, Tumor versus Control

Downregulated miRNAs			
Rank	Common name	Targets in test set^a^	Total targets in genome
1	hsa‐miR‐612	364	4140
2	hsa‐miR‐378c	308	2896
3	hsa‐miR‐422a	283	2599
4	hsa‐miR‐490‐3p	240	1971
5	hsa‐miR‐378b	242	2201
6	hsa‐miR‐339‐5p	200	2149
7	hsa‐miR‐1269	161	1851
8	hsa‐miR‐139‐3p^b^	53	578

Upregulated miRNAs			
Rank	Common name	Targets in test set	Total targets in genome
1	hsa‐miR‐450b‐5p	41	4508
2	hsa‐miR‐199a‐3p	29	2355
3	hsa‐miR‐199b‐3p	28	2355
4	hsa‐miR‐361‐5p	17	3005

^a^ Test set = differentially expressed mRNAs.

^b^ The opposite strand is the dominant form.

Although the role of these miRNAs in pediatric HCC remains largely obscure, many were consistent with available literature's observations in other types of cancer supporting the feasibility of our analysis. For example, miRNAs downregulated in pediatric HCC such as miR‐612, miR‐378c, miR‐422a, miR‐339‐5p, and miR‐378b (Table 2) play tumor‐suppressive functions in adult HCC and/or other types of cancer,^31^, ^32^, ^33^, ^34^, ^35^, ^36^, ^37^, ^38^, ^39^, ^40^, ^41^, ^42^, ^43^, ^44^, ^45^, ^46^, ^47^, ^48^, ^49^, ^50^, ^51^, ^52^ supporting the existence of a common mechanism involved both in adult and pediatric settings.

**FIGURE 1:**
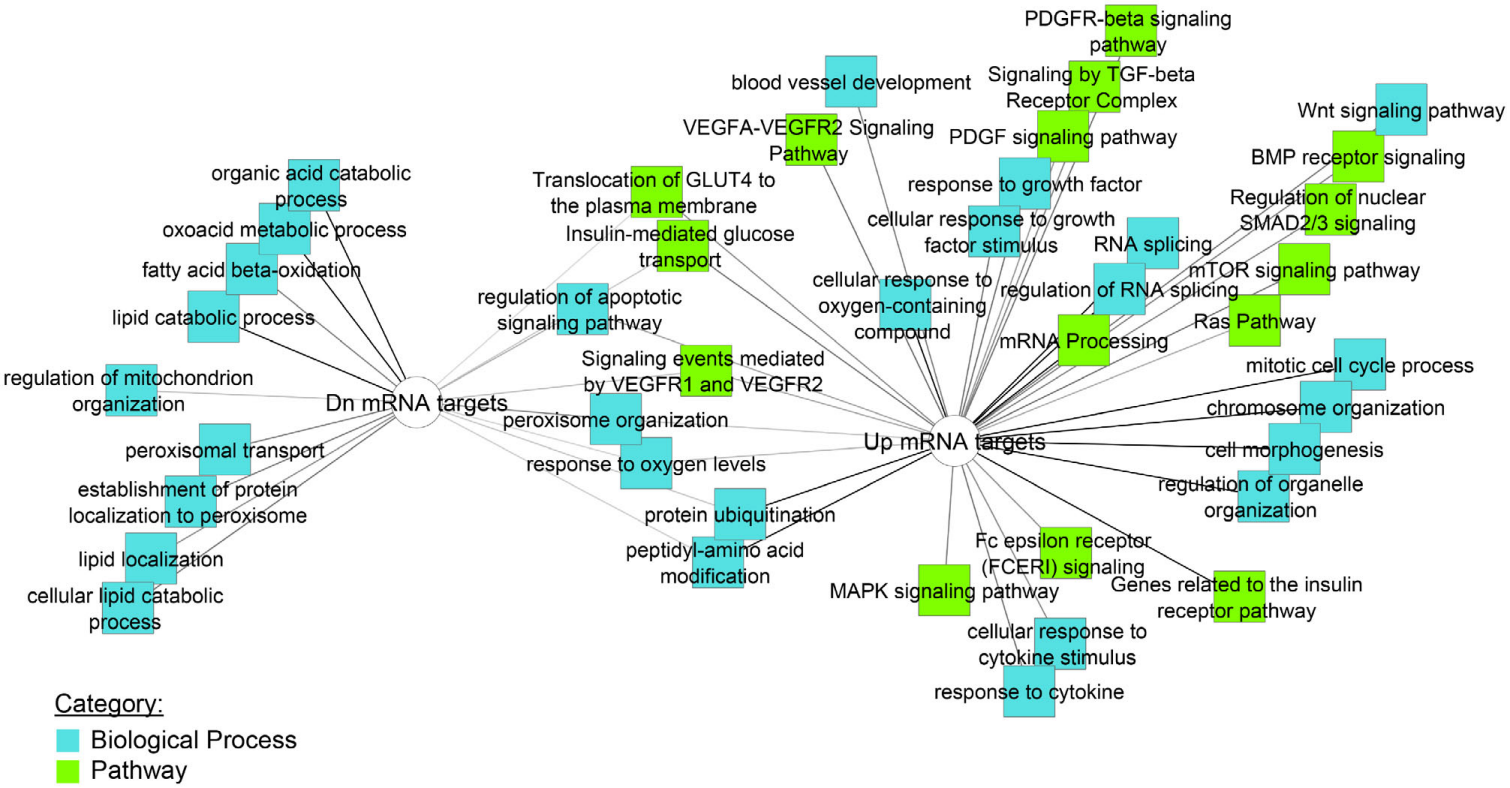
Ontology analysis of top‐ranked target mRNAs, Tumor versus Control. Analysis was performed using downregulated target mRNAs of upregulated miRNAs (left) and upregulated target mRNAs of downregulated miRNAs (right)

Ontology analysis of upregulated target mRNAs of top‐ranked downregulated miRNAs indicates activation of several biological processes associated with tumorigenesis such as “mitogen‐activated protein kinase (MAPK) signaling pathway,” “mitotic cell cycle process” “Wnt signaling pathway” “Ras Pathway,” “blood vessel development” and signaling pathways related to fibrosis such as “platelet‐derived growth factor (PDGF) signaling pathway” and “signaling by transforming growth factor (TGF)‐beta receptor complex” (Figure 1).^53^, ^54^, ^55^, ^56^, ^57^, ^58^ Processes associated with RNA splicing and processing were also enriched. Of note, altered mRNA processing has been correlated with liver cancer, and dysregulation of miRNAs is often associated with disturbance of the splicing process in many diseases.^59^, ^60^, ^61^, ^62^, ^63^ Our results suggest that several miRNAs are deregulated in pediatric HCC as compared to no disease controls and these miRNAs are upstream of biological processes involved in tumor development and progression.

### 3.3 Profiling of FLC identified overlapping and unique miRNAs deregulated in FLC

**FIGURE 2:**
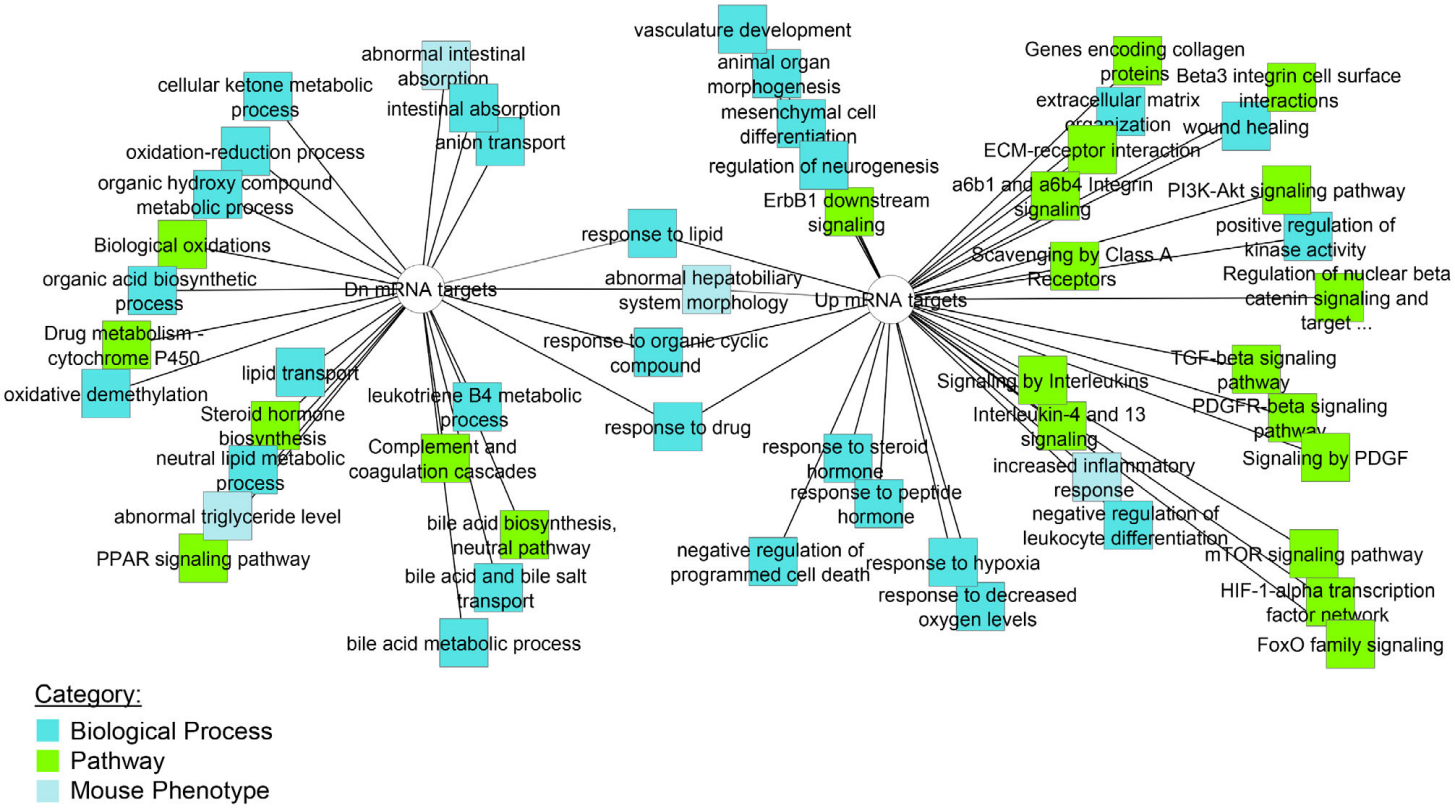
Ontology analysis of top‐ranked target mRNAs, fibrolamellar carcinoma (FLC) versus Control. Analysis was performed using downregulated target mRNAs of upregulated miRNAs (left) and upregulated target mRNAs of downregulated miRNAs (right)

As pediatric HCC is a heterogeneous group of diseases,^2^ we aimed to identify miRNAs whose levels are altered in a specific subset disease, FLC. PCA analysis of FLC tumors, non‐FLC tumors, and no liver disease control samples using miRNA expression revealed a higher magnitude of separation between FLC and control samples than between non‐FLC and controls (Figure S1D). While our analysis did not detect differentially expressed miRNAs between non‐FLC and FLC at statistically significant levels, we identified 59 downregulated and 77 upregulated miRNAs in FLC as compared to no liver disease controls (Table S3). ToppMiR was used to generate prioritized lists of miRNAs (Table 3) and target mRNAs (Table S7). A substantial overlap of top‐ranked miRNAs was found between the two miRNA sets, “Tumor versus Control” and “FLC versus Control,” suggesting common roles of these miRNAs regardless of histological subtypes (Figures S2 and S3). There were three downregulated miRNAs uniquely captured in “FLC versus Control” but not identified in “Tumor versus Control” and “BG versus Control”: miR‐548b‐3p, miR‐326, and miR‐375. miR‐375 is a well‐established tumor suppressor in FLC supporting the validity of our analysis.^64^ miR‐548b‐3p and miR‐326 are tumor suppressors in adult HCC and other cancers although their role in FLC remains to be determined.^65^, ^66^, ^67^, ^68^, ^69^, ^70^, ^71^, ^72^, ^73^, ^74^, ^75^, ^76^, ^77^, ^78^ Ontology analysis of upregulated target mRNAs of top‐ranked downregulated miRNAs indicates activation of processes reported to be altered in FLC including those involved in extracellular matrix, integrin signaling, response to hypoxia, and inflammatory response in addition to signaling pathways involved in oncogenesis and fibrosis (Figure 2).^79^, ^80^, ^81^, ^82^ Analysis of downregulated target mRNAs of top‐ranked upregulated miRNAs indicated that downregulated processes were those related to normal liver functions including lipid metabolism, bile acid metabolism, drug metabolism, oxidation–reduction process, and coagulation cascades.^83^, ^84^, ^85^, ^86^ Our data suggest overlapping and distinct roles of miRNAs in FLC as compared to non‐FLC tumors.

**TABLE 3:** Top‐ranked miRNAs, FLC versus Control

Downregulated miRNAs			
Rank	Common name	Targets in test set	Total targets in genome
1	hsa‐miR‐320c	282	4132
2	hsa‐miR‐320b	276	4141
3	hsa‐miR‐320a	277	4143
4	hsa‐miR‐320d	266	3972
5	hsa‐miR‐548b‐3p	215	2941
6	hsa‐miR‐28‐3p	148	1988
7	hsa‐miR‐378c	142	2896
8	hsa‐miR‐422a	132	2599
9	hsa‐miR‐326	152	3086
10	hsa‐miR‐769‐3p^a^	111	1857
11	hsa‐miR‐378	125	2739
12	hsa‐miR‐339‐5p	114	2149
13	hsa‐miR‐375	104	1287
14	hsa‐miR‐378b	110	2201
15	hsa‐miR‐671‐5p	110	2297
16	hsa‐miR‐1269	94	1951
17	hsa‐miR‐483‐5p	53	1088
18	hsa‐miR‐339‐3p	27	478
19	hsa‐miR‐139‐3p^a^	30	578
20	hsa‐miR‐127‐3p	18	471

Upregulated miRNAs			
Rank	Common name	Targets in test set	Total targets in genome
1	hsa‐miR‐590‐3p	115	8612
2	hsa‐miR‐543	74	4695
3	hsa‐miR‐450b‐5p	58	4508
4	hsa‐miR‐34c‐5p	78	3238
5	hsa‐miR‐576‐5p	53	4162
6	hsa‐miR‐628‐5p	47	2516
7	hsa‐miR‐299‐3p^a^	46	2224
8	hsa‐miR‐485‐3p^a^	49	3176
9	hsa‐miR‐361‐5p	33	3005
10	hsa‐miR‐199b‐3p	34	2355
11	hsa‐miR‐199a‐3p	34	2355
12	hsa‐miR‐337‐3p	29	2379
13	hsa‐miR‐299‐5p	31	2232
14	hsa‐miR‐337‐5p^a^	11	720
15	hsa‐miR‐369‐5p^a^	7	299

Abbreviation: FLC, fibrolamellar carcinoma.

^a^ The opposite strand is the dominant form.

### 3.4 Profiling of background livers identified miRNAs altered in both tumors and background liver tissues

To identify molecular events altered in non‐tumorous background liver tissues from patients with liver cancer, we analyzed differentially expressed miRNAs between BG versus Control and identified 80 downregulated miRNAs and 87 upregulated miRNAs (Table S4). We incorporated the list of differentially expressed mRNAs (Table S8) in ToppMiR analysis and established prioritized lists of miRNAs (Table 4) and potential target mRNAs (Table S9). Many downregulated miRNAs such as miR‐671‐5p, miR‐422a, and miR‐339‐5p act as tumor suppressors in adult HCC, hepatoma cell lines, and non‐hepatic cancers.^36^, ^37^, ^38^, ^39^, ^40^, ^41^, ^42^, ^43^, ^44^, ^45^, ^46^, ^47^, ^48^, ^49^, ^50^, ^87^ These miRNAs were also downregulated in FLC and/or Tumor (Figure S2), implying their involvement in the multiple stages of disease progression. Members of the miR‐320 family were the four most highly ranked downregulated miRNAs in “FLC versus Control” as well as in “BG versus Control” (Tables 3 and 4). While there is scant literature on miR‐320 s' role in pediatric liver cancer, their tumor‐suppressive functions are well established in other cancers.^88^, ^89^, ^90^, ^91^, ^92^, ^93^, ^94^, ^95^ miR‐484, which expression was upregulated in “BG versus Control” but not in Tumor or FLC (Figure S3), is involved in tumor initiation and precancerous lesion formation in a mouse model of liver cancer.^96^, ^97^, ^98^ Ontology analysis of target mRNAs indicates biological processes altered in Tumor and/or FLC are also affected in BG including mRNA processing, TGF and PDGF signaling, and lipid metabolism (Figure S4). Our results suggest that miRNAs deregulate several biological processes in not only tumor tissues but also corresponding non‐tumorous background liver tissues.

**TABLE 4:** Top‐ranked miRNAs, BG versus Control

Downregulated miRNAs			
Rank	Common name	Targets in test set	Total targets in genome
1	hsa‐miR‐320c	320	4132
2	hsa‐miR‐320b	314	4141
3	hsa‐miR‐320a	314	4143
4	hsa‐miR‐320d	304	3972
5	hsa‐miR‐409‐3p	314	3790
6	hsa‐miR‐612	226	4140
7	hsa‐miR‐28‐3p	175	1988
8	hsa‐miR‐455‐5p^a^	156	2149
9	hsa‐miR‐1260	140	2594
10	hsa‐miR‐320e	183	2635
11	hsa‐miR‐1275	142	2742
12	hsa‐miR‐378c	186	2896
13	hsa‐miR‐769‐3p^a^	125	1857
14	hsa‐miR‐574‐5p^a^	158	2530
15	hsa‐miR‐378	167	2739
16	hsa‐miR‐1260b	137	2410
17	hsa‐miR‐422a	163	2599
18	hsa‐miR‐1269	133	1951
19	hsa‐miR‐671‐5p	124	2297
20	hsa‐miR‐490‐3p	134	1971
21	hsa‐miR‐324‐3p^a^	140	2629
22	hsa‐miR‐378b	138	2201
23	hsa‐miR‐760	135	2791
24	hsa‐miR‐339‐5p	122	2149
25	hsa‐miR‐193a‐5p^a^	102	1916
26	hsa‐miR‐483‐5p	55	1088
27	hsa‐miR‐941	31	731
28	hsa‐miR‐3131	32	591
29	hsa‐miR‐139‐3p^a^	33	578
30	hsa‐miR‐339‐3p	19	478
31	hsa‐miR‐127‐3p	26	471

Upregulated miRNAs			
Rank	Common name	Targets in test set	Total targets in genome
1	hsa‐miR‐590‐3p	98	8612
2	hsa‐miR‐484	70	2472
3	hsa‐miR‐450b‐5p	56	4508
4	hsa‐miR‐628‐5p	41	2516
5	hsa‐miR‐429	35	4256
6	hsa‐miR‐802	44	3770
7	hsa‐miR‐590‐5p	33	2821
8	hsa‐miR‐199b‐3p	28	2355
9	hsa‐miR‐199a‐3p	29	2355
10	hsa‐miR‐576‐5p	39	4162
11	hsa‐miR‐139‐5p	32	2851
12	hsa‐miR‐146b‐5p	28	2987
13	hsa‐miR‐361‐5p	23	3005
14	hsa‐miR‐337‐3p	32	2379
15	hsa‐miR‐299‐5p	25	2232
16	hsa‐miR‐885‐5p	15	2181
17	hsa‐miR‐369‐5p^a^	6	299

^a^ The opposite strand is the dominant form.

## 4 DISCUSSION

The aim of the current study was to gain insight into the alteration and biological relevance of miRNAs in pediatric HCC, which remain largely obscure. To this end, we performed RNA sequencing on samples from FLC and non‐FLC pediatric HCC as well as corresponding non‐tumorous background liver tissues and no liver disease control liver tissues obtained from young patients. These results were grouped into three broad categories: Tumor versus Control, FLC versus Control, and BG versus Control. We then used ToppMiR to prioritize mRNAs based on their enriched terms and relative biological relevance and to score miRNAs according to the potential biological relevance and impact of their targets.^6^ Our analysis uncovered several miRNAs with altered expression, which roles are unclear in pediatric HCC but reported in adult HCC, hepatoma cell lines, or other non‐hepatic tumors. In addition, some of the miRNAs identified using our analysis including miR‐450b‐5p, miR‐199s, and miR‐361‐5p, whose expressions were upregulated in all three categories, may act as an oncomiR or a tumor suppressor in certain types of tumors although their functions are ambiguous in pediatric HCC, implying a contextual role of these miRNAs.^99^, ^100^, ^101^, ^102^, ^103^, ^104^, ^105^, ^106^, ^107^, ^108^, ^109^, ^110^, ^111^, ^112^, ^113^, ^114^, ^115^, ^116^, ^117^, ^118^, ^119^, ^120^, ^121^ Given the relatively small number of samples analyzed in the current study and the heterogeneous nature of clinical variables, future experimental investigation of candidate miRNAs would require a model system recapitulating a specific subtype of pediatric HCC. Of note, ToppMiR uses all seven sources of miRNA target predictions, two of which are based on experimentally validated targets^6^, ^12^, ^13^, ^14^, ^15^, ^16^, ^17^, ^18^, ^19^, ^20^, ^21^, ^22^, ^23^, ^24^, ^25^, ^26^; thus, targets identified by ToppMiR may not be from experimentally verified miRNA sources. Nevertheless, some of the experimentally validated targets in other types of cancers described in the references we are citing were also identified using ToppMiR, including forkhead box Q1 (*FOXQ1*) and proteolipid protein 2 (*PLP2*) (targets of miR‐422a),^36^, ^39^ transcription factor 4 (*TCF4*, a target of miR‐326),^77^ and S100 calcium binding protein A10 (*S100A10*) and neuropilin 1 (*NRP1*) (targets of miR‐320s)^89^, ^95^ (Tables S7 and S9). Another limitation of the current study that should be considered for future validation is that our analysis did not investigate protein levels although miRNAs not only induce mRNA degradation but also inhibit translation.^122^

Our PCA analysis indicated a smaller magnitude of differences between the non‐FLC and control samples compared to the FLC and control samples. Statistically, the higher degree of variability among the non‐FLC samples may be obscuring some biological signal, which leaves the “Tumor versus Control” analysis being driven primarily by the FLC samples. This explains why most miRNAs differentially regulated in “Tumor versus Control” were also differentially regulated in “FLC versus Control”, and the lack of any significant miRNAs in the “non‐FLC versus Control” analysis.

Ontologies of target mRNAs were mostly within expectations for upregulated biological processes, with most of these being related to proliferation, migration, invasion, and response to growth factors. For both the FLC versus Control and BG versus Control groups, however, there was a significant downregulation of several metabolic functions of the liver including lipid metabolism and oxidation–reduction process. While our data seem to contradict the fact that cancer cells often exhibit increased anabolism to support proliferation and migration,^123^ metabolic dysregulation has been reported in the context of adult HCC and other types of cancers.^53^, ^83^, ^123^, ^124^, ^125^, ^126^, ^127^, ^128^, ^129^, ^130^ Whether altered metabolism is a consequence of hepatocyte de‐differentiation or causal to tumorigenesis warrant further investigation.

In summary, our investigation led to significant discoveries that: (a) several miRNAs with altered expression in pediatric HCC or corresponding liver tissues were reported to regulate tumorigenesis in other types of tumors, implying a common role of these miRNAs in multiple contexts; (b) analysis of background liver tissues suggest roles of miRNA–mRNA networks from the early step of disease progression; (c) ToppMiR‐based analysis reveals roles of miRNAs and their targets not only in biological processes related to tumor initiation and progression but also metabolic functions of the liver. Each identified miRNA and corresponding targets may serve as a promising subject of mechanistic investigation and potential therapeutic tool in the treatment of pediatric HCC.

## AUTHOR CONTRIBUTIONS

**Senyo S Whyte:** Conceptualization (equal); data curation (equal); investigation (equal); writing – original draft (lead); writing – review and editing (equal). **Rebekah Karns:** Data curation (equal); formal analysis (equal); visualization (equal); writing – review and editing (equal). **Kyung‐Won Min:** Formal analysis (equal); investigation (equal); writing – review and editing (equal). **Jung‐Hyun Cho:** Formal analysis (equal); investigation (equal); writing – review and editing (equal). **Sanghoon Lee:** Data curation (equal); investigation (equal); writing – review and editing (equal). **Charissa Lake:** Data curation (equal); writing – review and editing (equal). **Alexander Bondoc:** Data curation (equal); investigation (equal); writing – review and editing (equal). **Je‐Hyun Yoon:** Formal analysis (equal); investigation (equal); writing – review and editing (equal). **Soona Shin:** Conceptualization (equal); investigation (equal); supervision (lead); visualization (equal); writing – original draft (supporting); writing – review and editing (equal).

## CONFLICT OF INTEREST

The authors have stated explicitly that there are no conflicts of interest in connection with this article.

## ETHICS STATEMENT

The study protocol and informed consent forms were approved by the Institutional Review Board of the Cincinnati Children's Hospital Medical Center.

## Supporting information


**FIGURE S1** (A‐C) Heat maps of miRNAs differentially expressed between (A) Tumor versus Control, (B) FLC versus Control, and (C) BG versus Control. (D) PCA analysis of FLC and non‐FLC tumors versus Control.
**FIGURE S2**. Venn diagram of top‐ranked miRNAs downregulated compared to the control group.
**FIGURE S3**. Venn diagram of top‐ranked miRNAs upregulated compared to the control group.
**FIGURE S4**. Ontology analysis of top‐ranked target mRNAs, BG versus Control. Analysis was performed using downregulated target mRNAs of upregulated miRNAs (left) and upregulated target mRNAs of downregulated miRNAs (right).

TABLE S1 List of samples included in datasets
**TABLE S2**. Differentially expressed miRNAs in Tumor versus Control
**TABLE S3**. Differentially expressed miRNAs in FLC versus Control
**TABLE S4**. Differentially expressed miRNAs in BG versus Control
**TABLE S5**. Differentially expressed mRNAs in Tumor versus Control
**TABLE S6**. Top‐ranked mRNA targets, Tumor versus Control
**TABLE S7**. Top‐ranked mRNA targets, FLC versus Control
**TABLE S8**. Differentially expressed mRNAs in BG versus Control
**TABLE S9**. Top‐ranked mRNA targets, BG versus Control

## Data Availability

The data that support the findings of this study are available from the corresponding author upon reasonable request. The data are not publicly available due to privacy or ethical restrictions.
