# Supplementary material for: Integrated analysis using ToppMiR uncovers altered miRNA– mRNA regulatory networks in pediatric hepatocellular carcinoma—A pilot study
Source: Cancer Rep (Hoboken). 2022 Jul 20;6(1):e1685. doi: 10.1002/cnr2.1685 (PMC9875636; doi:10.1002/cnr2.1685)
Supplement: Supplementary file 1 — FIGURE S1 (A‐C) Heat maps of miRNAs differentially expressed between (A) Tumor versus Control, (B) FLC versus Control, and (C) BG versus Control. (D) PCA analysis of FLC and non‐FLC tumors versus Control. FIGURE S2. Venn diagram of top‐ranked miRNAs downregulated compared to the control group. FIGURE S3. Venn diagram of top‐ranked miRNAs upregulated compared to the control group. FIGURE S4. Ontology analysis of top‐ranked target mRNAs, BG versus Control. Analysis was performed using downregulated target mRNAs of upregulated miRNAs (left) and upregulated target mRNAs of downregulated miRNAs (right). [file CNR2-6-e1685-s001.pdf]

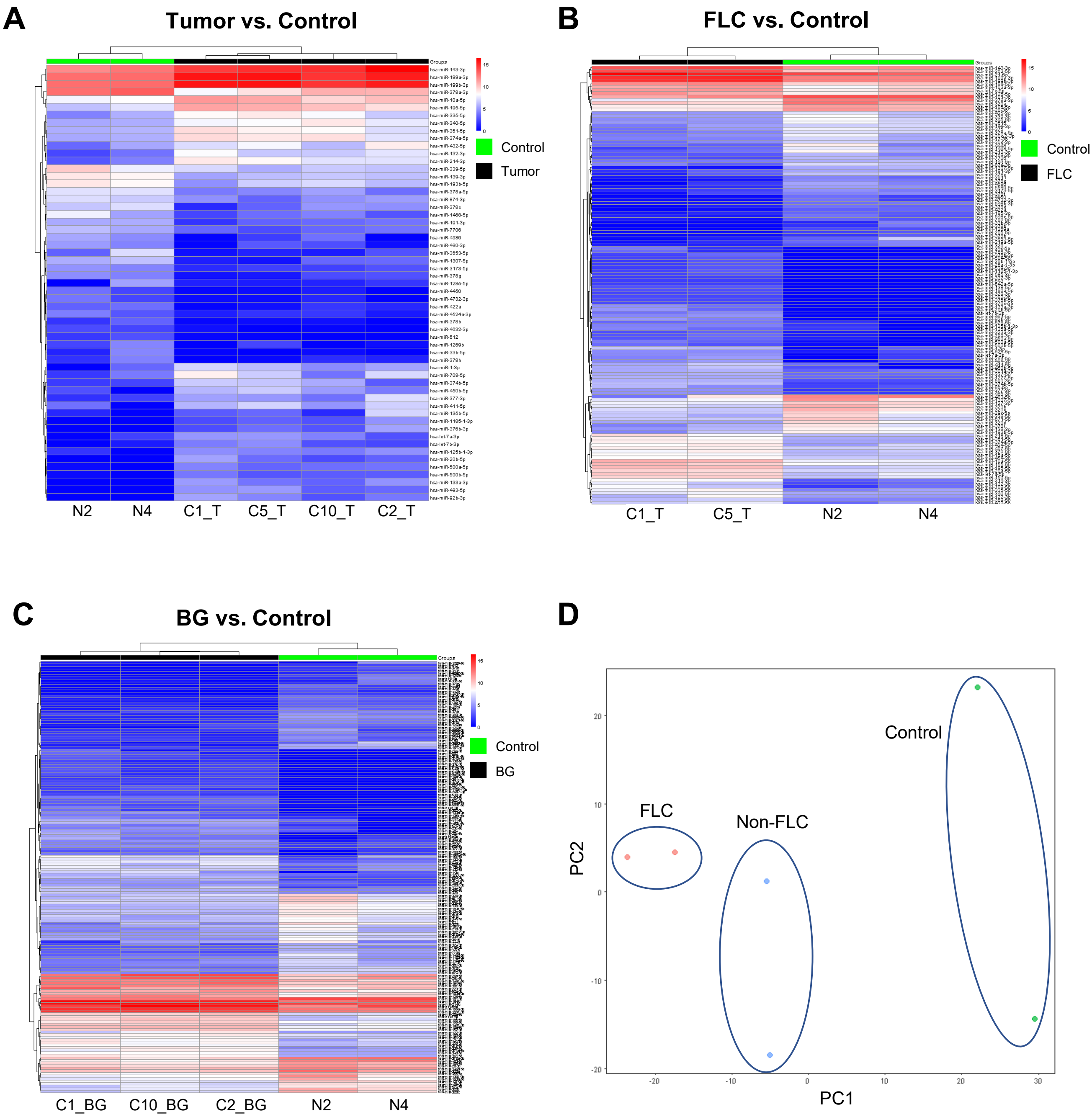

SUPPORTING INFORMATION FIGURE S1. (A-C) Heat maps of miRNAs differentially expressed between (A) Tumor vs. Control, (B) FLC vs. Control, and (C) BG vs. Control. (D) PCA analysis of FLC and non-FLC tumors vs. Control.

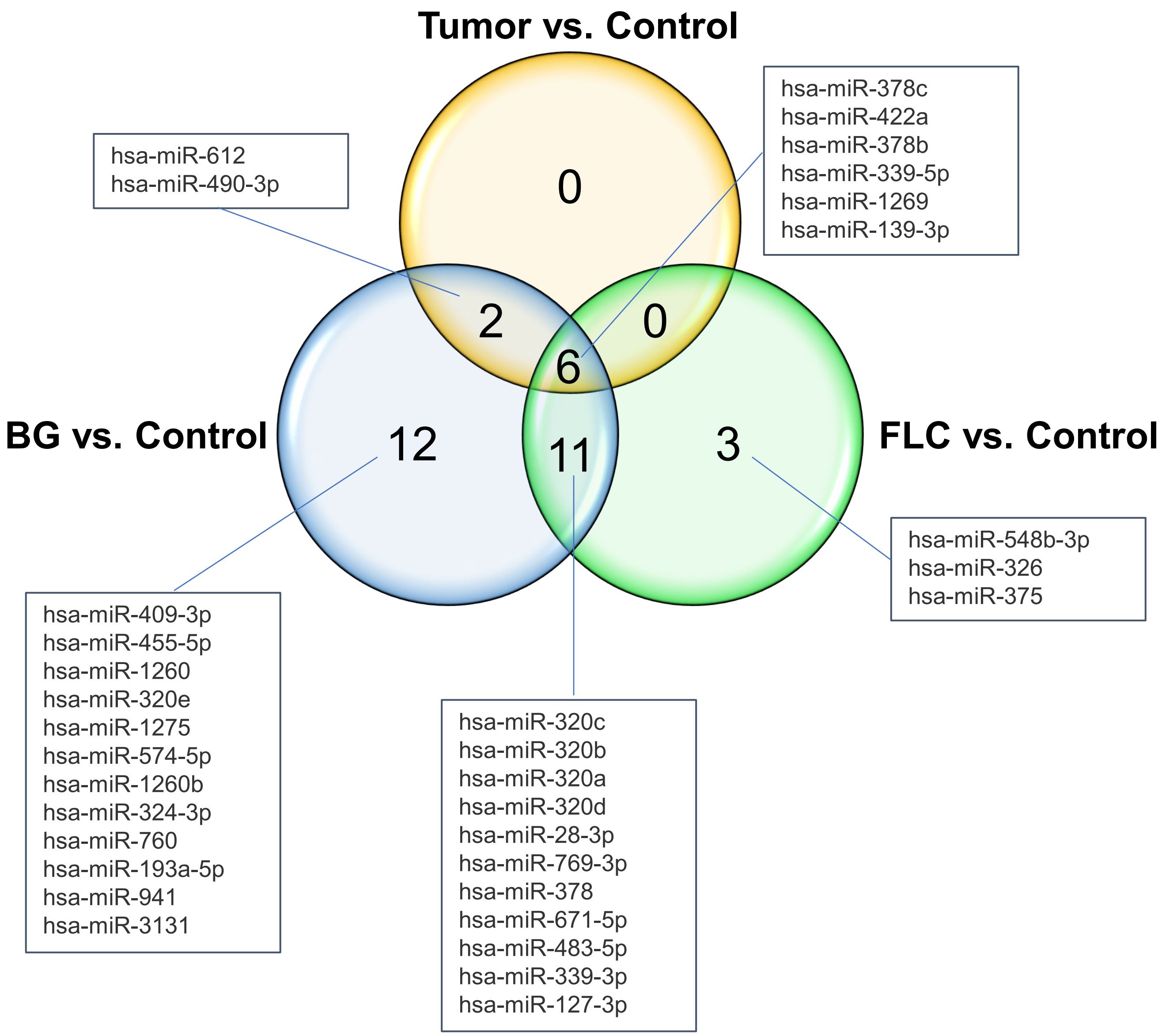

SUPPORTING INFORMATION FIGURE S2. Venn diagram of top-ranked miRNAs downregulated compared to the control group.

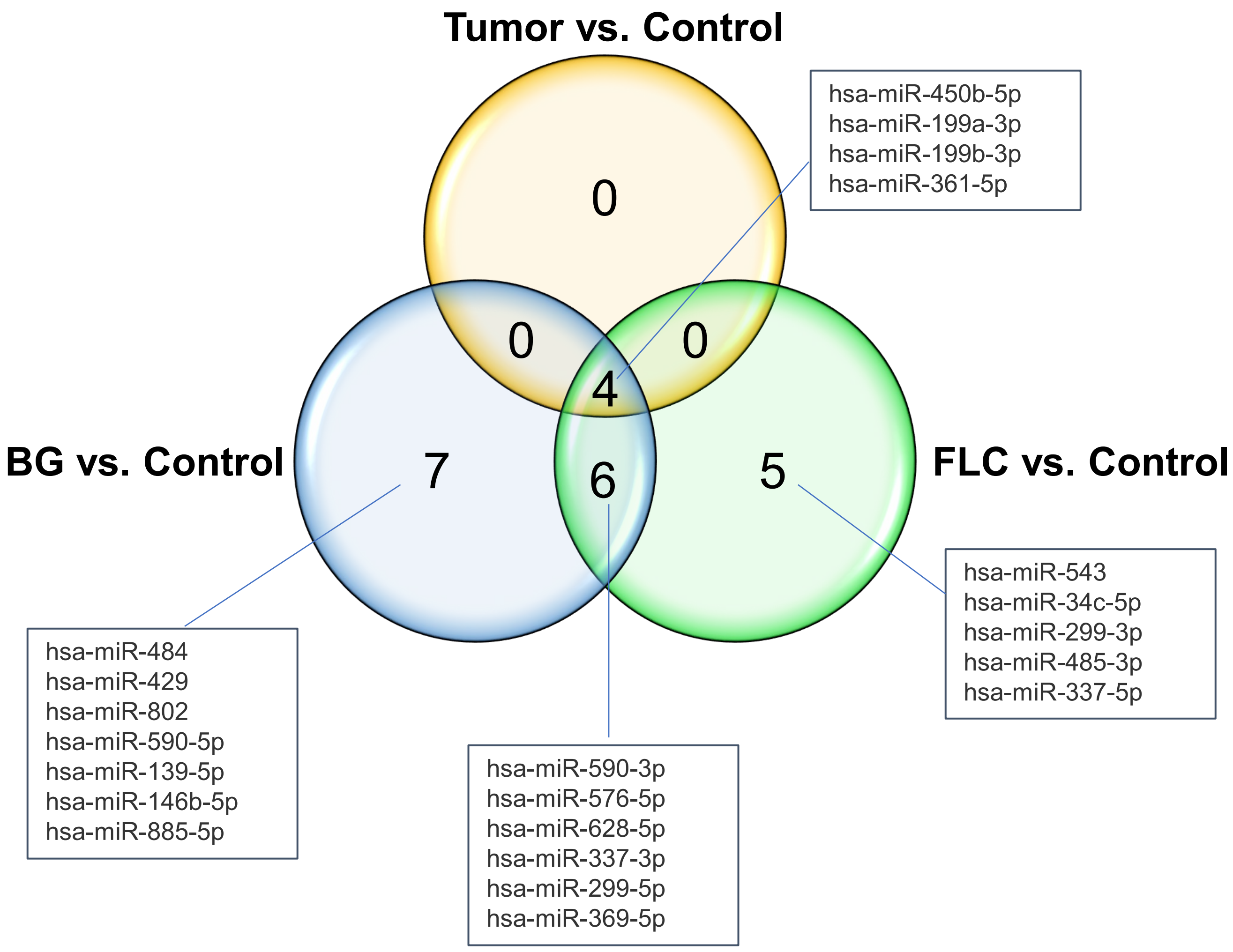

SUPPORTING INFORMATION FIGURE S3. Venn diagram of top-ranked miRNAs upregulated compared to the control group.

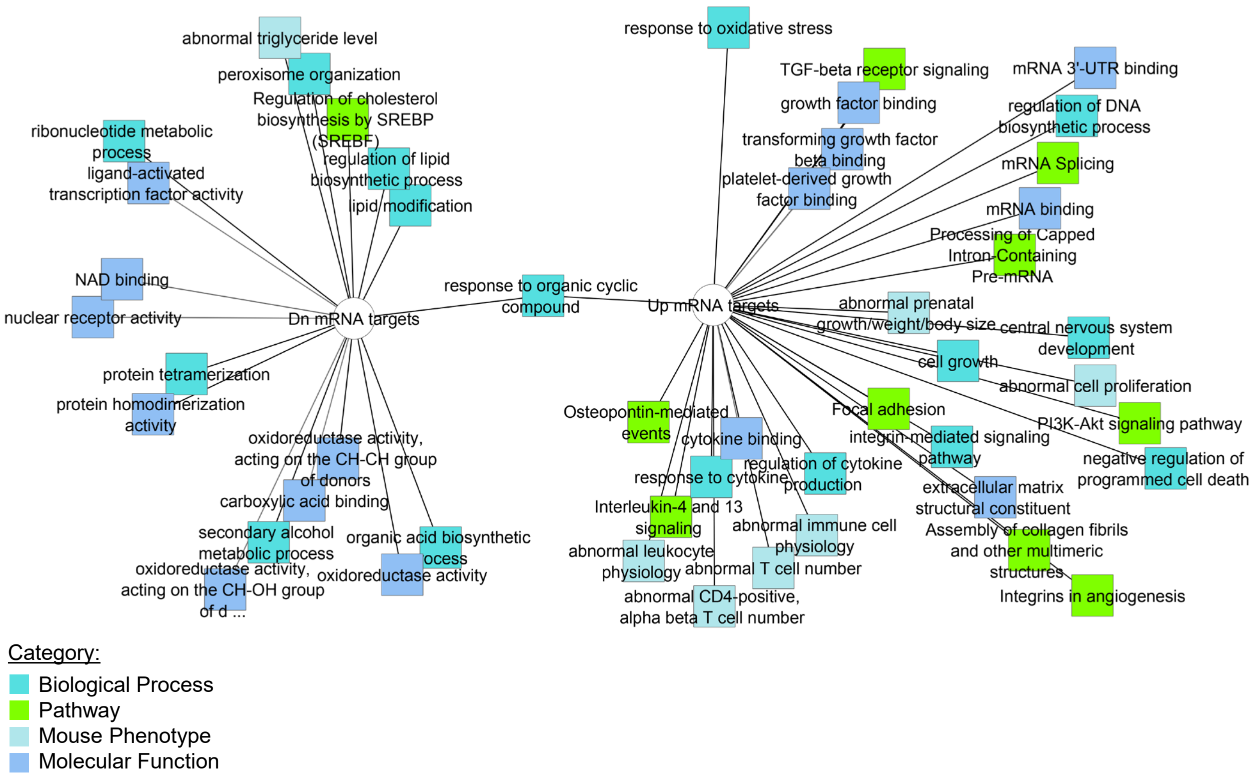

SUPPORTING INFORMATION FIGURE S4. Ontology analysis of top-ranked target mRNAs, BG vs. Control. Analysis was performed using downregulated target mRNAs of upregulated miRNAs (left) and upregulated target mRNAs of downregulated miRNAs (right).
